# Supplementary material for: Are carbon emissions trading and green financial instruments synergistic? -Comprehensive quantitative research based on content analysis
Source: PLoS One. 2024 Mar 7;19(3):e0298601. doi: 10.1371/journal.pone.0298601 (PMC10919601; doi:10.1371/journal.pone.0298601)
Supplement: S1 Data — (ZIP) [file pone.0298601.s001.zip › Data/地级市Z_F工作报告.zip!/║┌┴·╜¡/║┌║╙╩╨/└┤╫╘╣1⁄2╓┌║┼í╛┬φ┐╦╩2╛▌═°í┐_2014.docx]

各位代表：

现在，我代表市人民政府，向大会作工作报告，请予审议，并请列席会议的同志提出宝贵意见。

一、2013年工作回顾

2013年，在省委、省政府和市委正确领导下，我们坚持生态发展，实现绿色崛起，打好“三大攻坚战”，实施“五大工程”，经济社会持续健康发展，较好地完成了预期目标。全市生产总值实现389.6亿元，增长8.1%。全社会固定资产投资269.6亿元，增长26%。社会消费品零售总额84.2亿元，增长14.6%。公共财政收入23.1亿元，增长11.8%，其中，市直公共财政收入6.9亿元，增长22%。对外贸易进出口总值41.9亿美元，增长10.1%。城镇居民人均可支配收入17817元，农村居民人均纯收入10690元，分别增长10.1%和10.2%。万元地区生产总值综合能耗下降3.2%。

去年入汛后，我市遭受1984年以来最大的洪涝灾害，黑龙江黑河段经历4次洪峰，嫩江为历史最大洪水。面对严峻形势，我们把确保人民生命安全放在首位，周密部署，科学指挥，与广大基层干部群众、解放军、武警、预备役官兵共同奋战，夺取了抗洪抢险的全面胜利，实现了省委、省政府提出的“江河不溃堤、水库不垮坝、人员不伤亡”目标，保卫了国家和人民生命财产免受重大损失。超前启动灾后重建，抢排积水保粮食、抢建房屋保越冬、抢修设施保运转、抢抓项目保增长，9月中旬完成农田排涝273万亩，10月中旬超额完成房屋重建计划，修复重建5252户，水毁设施修复工程有序推进，全市在短时间内恢复了正常生产生活秩序。在这场抗洪抢险斗争中，党政军民携手并肩、同舟共济，熔铸成伟大的抗洪精神，成为未来推进黑河各项事业发展的强大动力！

一年来，我们主要做了以下工作：

(一)应对困难挑战，内生动力持续增强。针对自然灾害和市场低迷双重考验，科学研判，有效应对，对内挖掘潜力，对外赢得支持。“六大规划”带来新机遇。经积极争取，先后纳入国家“六大规划”。黑龙江和内蒙古东北部地区沿边开发开放规划、中国东北地区面向东北亚区域开放规划，赋予我市对外开放先行先试政策。北安、嫩江、五大连池纳入黑龙江省“两大平原”现代农业综合配套改革试验区，爱辉区、孙吴、逊克参照享受政策扶持。资源型城市可持续发展规划将我市确定为成熟型地市级资源城市，得到国家政策支持。全市纳入大小兴安岭生态保护与经济转型规划，享受转移支付17.3亿元，专项扶持资金8000万元。黑河市(含爱辉区)、孙吴、逊克纳入兴边富民行动规划，享受西部大开发政策，争取资金3600万元。“三年决战”胜利收官。三年实施市级重点产业项目138项，完成投资237亿元，年均增长31%。省重点推进产业项目48项，完成投资160亿元，年均增长25.8%。产业项目投资占固定资产投资比重由40.3%提高到58.3%。2013年实施市级重点产业项目74项，完成投资100亿元，增长28.5%。省重点推进产业项目开复工28项，完成投资62.8亿元，增长12%。围绕“五大产业”，谋划储备项目500项，总投资1800亿元。明确园区发展定位，制定发展异地经济方案，因地制宜摆放项目，园区特色更加鲜明。实施中小企业成长工程，设立发展专项资金，全市规模以上工业企业由85户增加至96户，增加值增长16.7%。招商引资取得突破。创新招商机制，实施全员招商，认真开展“招商之冬”、“开工之春”、“集中签约”活动，在冬季成功举办大黑河岛国际经贸洽谈会，积极参加省内外大型经贸洽谈活动，宣传推介，主动对接，落实项目243项，到位资金185亿元，增长48.2%。

(二)培育“五大产业”，发展质量不断提升。坚持实业立市，立足区位资源优势，培育特色鲜明的主导产业。农林产业稳步发展。克服了春涝、低温、洪水等严重自然灾害，粮食在大灾之年再获丰收，农业增加值增长5.6%。田间综合机械化程度96.8%。全市农民专业合作社达到1051个，增长27.2%。孙吴桦林农机合作社晋升为省级规范社。土地承包经营权流转面积达到669万亩，增长15.4%，全市1200万亩适宜耕地全部实施规模经营。深入推进局市共建，“三代”面积918.5万亩，共建优质农产品生产基地900万亩。加快养殖基地建设，新增2个省部级标准化畜牧示范场。培育龙头企业，北安谷神大豆深加工、五大连池圣丰种业、孙吴爱俐华精制糖等项目加快建设。推进林业产业发展，蓝莓、沙棘等小浆果基地规模不断扩大，北药种植达到42.6万亩。黑河农业科技园区晋升为国家级。矿产开发有序推进。深入实施“矿业富市”战略，矿业经济产值、税收分别增长35.1%和59.7%。加快地质找矿，引进中铝力拓公司进行整装勘查。实施“1456”矿产项目计划，争光岩金矿、铜山铜矿改扩建、翠宏山铁多金属矿等项目前期进展顺利。加强煤矿整治整合，永发、同兴等煤矿开工。建设绿色矿山，启动煤矿采空区、沙金过采区综合治理。文化旅游融合发展。第四届中俄文化大集上升为中俄两国文化交流机制化项目，影响力进一步提升。成功举办鄂伦春族下山定居60周年庆祝活动、五大连池火山圣水节。博物馆集群发展，北安博物馆、庆华军工遗址博物馆和省委、省政府旧址陈列馆建成开放，嫩江古道驿站博物馆完工。旅游产业提档升级，编制了旅游业发展总体规划、中俄双子城旅游业发展规划。五大连池风景区等景区基础设施不断完善。承办了第三届中俄地区旅游合作交流会，“璀璨双子城、异国圣诞节”主题旅游活动受到广泛关注，开展了“4+1”城际旅游联盟促销活动。积极开发黑河—伊春、黑河—漠河等旅游线路，首届黑龙江黑河—俄罗斯贝加尔湖国际自驾游，荣获2013中国体育旅游十佳精品线路。对俄合作进一步拓展。优化贸易结构，首次实现燃料油进口。油品储运、奥格贾煤矿等一批对俄合作项目取得积极进展，云津和首钢集团境外铁矿煤矿开发与俄方达成合作意向。开通了黑龙江浮箱固冰客运通道。黑龙江大桥、跨江空中索道项目得到国家、省高度关切和大力支持。成功举办第六届中俄林业生态建设学术论坛。新兴产业加速成长。大力发展新能源及装备制造、生物医药、现代信息等战略性新兴产业，北安上海风电设备制造二期、爱辉区小江生物制药、合作区聚乙烯醇大化工等项目建成，嫩江生物质发电等项目加快建设，云计算产业开始起步。

(三)推进城乡统筹，发展基础更加坚实。加大投入力度，推进城乡一体化发展。城市建设管理水平不断提升。“三城同创”扎实推进，国家卫生城创建取得突破。加快市政工程建设，中央街、西兴路等街路完成升级改造，输水复线、热网改造二期、污水处理厂二期完工，污泥处理厂项目启动。新增立体景观15处、绿地4.2万平方米。加大环卫设施投入，垃圾无害化处理率80%，机械化清雪率70%。数字地理空间框架项目投入运行，提升了城市管理信息化水平。新型城镇化扎实推进。坚持试点带动，强化产业支撑，全市15个城镇化试点乡(镇)开工基础设施、公共服务设施、产业园区项目45项，完成投资7.2亿元，城镇吸纳能力显著增强。探索创新户籍管理和社保制度，放宽“农转非”落户政策，促进农民向城镇聚集。完成“三带百村工程”示范带30个重点村和47个现代农业示范园区建设。

承载能力显著提升。加大生态建设力度，造林绿化39.7万亩，森林抚育53.5万亩。实施“一退三还”行动，完成退耕还林、还草、还湿20.2万亩。嫩江中央站黑嘴松鸡自然保护区晋升国家级，五大连池山口湖纳入国家江河湖泊生态环保规划。加快水利工程建设，大黑河岛防护治理工程获得国家批准，逊克堤防水毁修复工程开工。争取水利建设资金10.1亿元，是我市水利建设史上投资最多、工程规模最大的一年。

(四)保障改善民生，社会保持和谐稳定。践行执政为民理念，全市民生支出76.5亿元，占公共财政支出57.7%，提高6.7个百分点。“十七件民生实事”完成15件，继续推进2件。人居环境持续改善。改造棚户区5224户、农村泥草房1.6万户、农村危房5100户，建设廉租住房910套，改造旧住宅小区40个。就业形势总体稳定。全市城镇新增就业2万人，就业困难人员实现就业5000人，城镇登记失业率3.7%，30周岁以下高校毕业生就业率85%。发放小额担保贷款1.6亿元，就业技能培训1.6万人。社会事业协调发展。新建改扩建公办幼儿园25所，创建市级一类幼儿园9所，省级标准化学校11所。启动了村卫生所标准化、急诊急救中心等90个医疗卫生项目。打造“动感城市”，承办了全国骑游文化展示大会、全省冰雪汽车挑战赛和中俄篮球赛。社会保障扩面提标。建立了覆盖城乡各类群体的社会保险和社会救助制度。各县(市、区)城镇职工、居民医疗保险最高支付限额均提高到6万元以上，市直达到8万元和6万元。提高了企业退休人员待遇，月人均增加养老金243元。失业和工伤保险实现市级统筹。在全省率先实施“先诊疗、后结算”新型医疗服务模式。新农保参保新增6.9万人。社会管理得到加强。全市养老机构达到60家，市失能老人养护中心、老年活动中心投入使用，民政福利园配套设施完工。社区建设走在全省前列，建设1000平方米以上城市示范社区12个，500平方米以上农村示范社区12个。北安、爱辉区城市社区全部开展居家养老服务。开展暖心行动，为市区街路保洁员免费提供“阳光早餐”。社会环境安定有序。推进煤矿井下安全避险系统建设，建立道路运输动态监控系统，安全生产形势稳定好转。连续15个防期未发生重特大森林火灾。食品药品市场秩序进一步规范。开展机关干部带案下访活动，信访形势总体平稳。社区矫正全面铺开。实施“军警民大巡防”行动，社会治安持续良好，边境管理进一步加强。人口计生、外事侨务、民族宗教、妇女儿童、防震减灾、地方志、人防等工作取得新成绩。

一年来，我们履职尽责，全面加强政府自身建设。主动接受人大依法监督和政协民主监督，144件建议和提案全部办结。完善重大决策专家咨询论证和公众参与机制，强化社会风险评估，重大决策审查率100%。推进“两集中、两到位”行政审批职能归并改革，优化审批流程，压缩办结时限。加大作风整治力度，出台了改进工作作风、提高行政效能实施细则，坚持深入基层调研，切实解决实际问题。加强应急预案演练，各类突发公共事件得到有效处置。完善权力运行监督机制，政府部门廉政风险防控实现全覆盖。

一年来的发展实践证明，我们经受住了困难和风险的考验，在科学发展的道路上又迈出了坚实步伐。这是省委、省政府和市委正确领导的结果，是人大、政协和社会各界鼎力支持的结果，是全市人民团结一致、共同奋斗的结果。在此，我代表市人民政府，向各级人大代表、政协委员和各民主党派、工商联、无党派人士、人民团体，向关心支持地方工作的中省直单位和人民解放军、武警官兵，向全市人民，表示衷心感谢，并致以崇高敬意！

二、2014年工作思路

今年是全面深化改革的开局之年，是实施“十二五”规划攻坚之年。我们要全面贯彻党的十八大、十八届三中全会精神，落实中央、省委工作部署，按照市委五届六次全会要求，围绕“六大规划”，坚持稳中求进和改革创新，突出经济发展和惠民利民，进一步扩总量、优结构、转职能，为生态发展、绿色崛起奠定坚实基础。

黑河仍处在欠发达阶段，加快发展仍是首要任务，需要我们根据区位条件、资源禀赋和发展实际，科学审视市情。应该看到，我市长期积累的矛盾和发展中出现的新问题相互交织，经济结构不合理的状况没有得到根本改变，三次产业比重47.9:17.5:34.6，农业大而不强，工业严重短板，消费业态原始。要明确主攻方向，推进三次产业协调健康发展。一产要做精做深，突出寒地黑土和绿色有机，大力发展绿色食品产业和畜牧业。二产要做大做强，坚持有所为有所不为，加快矿产资源开发，壮大战略性新兴产业。三产要做活做新，开发消费潜力，培育文化产业，发展试车、养老、碳汇和珠宝玉石“新四型经济”。

宏伟蓝图变成美好现实，方向比速度更重要。我们一定要以科学发展观为指导，顺势而为、乘势而上，在新的起点上赢得主动、赢得优势、赢得未来。牢牢把握以下理念：

——深化改革，实现创新型发展。改革激活力，创新无止境。要勇于突破思想禁锢，着力破解机制弊端，以更大的决心和智慧改革攻坚，用改革的精神、创新的思维、管用的办法促发展、谋转型，为经济社会发展提供强大动力。

——彰显特色，实现差异化发展。高度决定视野，眼界决定境界。树立全市一盘棋思想，科学确定各县(市、区)功能定位，加强与市域内农垦、森工、中省直企业联系沟通，共建共享、联合作战，构建百花齐放、优势互补的区域协调发展新格局。

——突出民生，实现福祉性发展。人民对美好生活的向往，就是我们的奋斗目标。要优化民生资源配置，加大财政对民生投入，实现和维护好群众的根本利益，让孩子们快乐成长，青年人、中年人、老年人都有希望、有憧憬，生活有尊严、日子有奔头。

——注重长远，实现积累式发展。千里始足下，高山起微尘。树立功成不必在我的思想境界，坚持符合黑河实际、被实践证明行之有效的发展思路，不换频道不换调，一张蓝图绘到底，通过一年一年的不懈努力，使经济社会始终沿着科学发展的方向阔步前行。

各位代表，改革发展是时代强音，强市富民是群众所盼。我们要以敢于担当的勇气，改革创新的精神，真抓实干的作风，坚定信心，迎难而上，在迎接挑战中把握机遇，在竞相发展中跨越赶超，奋力谱写更优、更美的发展新篇章！

三、2014年目标任务

今年，要保持工作连续性，继续打好“三大攻坚战”，实施“五大工程”，持之以恒、锲而不舍，在制约发展的重点领域和关键环节实现新突破。主要预期目标是：全市生产总值增长10%。全社会固定资产投资增长26%。规模以上工业增加值增长15%。社会消费品零售总额增长13.5%。公共财政收入增长11%。对外贸易进出口总值增长8%。城镇居民人均可支配收入、农村居民人均纯收入均增长10%。万元地区生产总值综合能耗下降3.2%。着重抓好以下工作：

(一)打好产业项目攻坚战。启动新一轮产业项目建设，围绕“五大产业”上项目，以资源立产业，以增量调结构，以发展促转型。全市安排重点产业项目80项，年度投资124亿元，增长18%。抓谋划生成。编制“五大产业”发展潜力报告。加强与大专院校、科研院所和龙头企业对接，谋划一批牵动力强、科技含量高的大项目。完善各部门、各县(市、区)项目谋划生成机构，设立招商科室的9个市直部门新谋划项目60项，各县(市、区)新谋划项目270项，50%要达到可研、项目建议书程度。抓开工投产。加大重点产业项目推进力度，投资5亿元以上项目由市级领导专项包保，每个项目要成立一个推进组，其他项目由县(市、区)负责推进。对计划新开工项目，千方百计落实要素保障，促进项目尽早开工。对已开复工项目，加强督导检查，力争建成投产率50%以上。抓园区建设。优化项目布局，增强项目选择与园区定位契合度。严格项目准入，不突破政策法规，不上产能过剩项目，不破坏生态环境，不减免应收的税费，不浪费可用的土地。入园项目固定资产投资不低于3000万元，高新技术和对俄进出口加工项目不低于1500万元。加快黑河进出口加工产业园、黑河(北安、孙吴)健康产业园、黑河国家农业科技园等特色园区建设。抓招商引资。突出重点产业和对俄经贸合作招商，提高签约项目落地率和资金到位率，到位资金增长30%。加强与国内外500强和行业领军企业战略合作，引导企业、商会和行业协会以商招商，在北京、上海、深圳等重点区域驻点招商。抓协调服务。严格落实产业项目责任制，坚持市县共同推进和集中会办，协调解决项目审批、落地、开工和建成投产等难题。继续实施中小企业成长工程，出台扶持政策，推动优质企业在“新三板”上市。鼓励金融机构助力中小企业发展，为企业投放贷款增长12%以上。积极向上争取，到位资金增长10%以上。

(二)打好重大基础设施建设攻坚战。争取政策支持，加大推进力度，为未来发展打好坚实基础。推进重大交通项目。争取黑龙江大桥、跨江空中索道项目前期有实质性进展。力争五大连池民用机场开工建设。争取恢复上海航线，旅游旺季增加至哈尔滨航班密度。沾五公路升级改造完工，国省干线水毁公路修复工程开工，升级改造逊克至逊孙界公路，新建嫩(泰)高速公路嫩江互通项目，争取北五铁路列入国家建设计划。实施重点水利工程。完成全市11条小河流、6座水库、5个主要涝区及逊克8公里堤防水毁修复工程，续建黑河城区、孙吴城区堤防工程。力争大黑河岛防护治理工程、黑龙江干流38公里堤防开工。实施西沟水库、北安红星水库除险加固及通肯河治理工程。西沟、象山等4个水电站增效扩容。争取黑龙江、嫩江干流未达标堤防全部纳入国家计划。完善农田水利设施。建设节水灌溉高标准农田，推进北安东胜、孙吴兴北、逊克高滩、爱辉区宋集屯等10处灌区建设，新增农田有效灌溉面积10.7万亩。

(三)打好城市建设攻坚战。围绕“三城同创”，坚持新城区与旧城区并举，地上与地下并重，建设与管理并进，优化人居环境。高水平做好规划。依据新一轮城市总体规划，坚持高起点、高标准，编制控详规划和修详规划，提高规划层次。打造中俄风情浓郁的滨江生态口岸城市，构筑“西山、北水、东湿地”城市生态格局，建设“百里界江、万顷湿地、山水环绕、一线多岛”沿江生态长廊。完善城市功能。启动浮箱固冰通道连接路和四中北侧道路建设，实施王肃街东段路灯安装工程，维修超期楼体亮化设施，完成黑龙江公园、大黑河岛植物园基础设施维修和改造工程。续建饮用水水源地污染防治项目，启动水厂工艺改造工程，改造兴林街、文化街、环城路供水干线，完成污泥处理厂建设，雨水防洪泵站、污水处理厂二期投入运营。改造黑河火车站及站前广场，设置主题雕塑。改造老旧城区。全面启动南部棚户区改造，争取3—5年完成1070户回迁任务。力争开工4个回迁区和土产路、规划三街。加快西部棚户区沿主干道拆迁改造，重点改造两块三角地和双拥大街北侧沿江棚户区，完成3条规划路连接线。开展绿化大会战。市区新增绿地25.9万平方米，改造绿地8.1万平方米。做精城市“绿点”，完善龙滨路与中央街、魁星路与龙科街等街路转角绿化，设置木质花箱、灯悬垂吊立体景观。延伸城市“绿线”，实施南出口、王肃街等16条街路绿化。扩大城市“绿面”，完成黑龙江公园四期和堤顶护坡绿化工程，启动中俄双子城公园建设。提高管理水平。开展“城市精细管理提升年”活动，实施户外广告、建设工地等“八项整治”，重点向背街小巷、居民社区和城乡结合部延伸。建立市直与爱辉区齐抓共管、多部门联动机制，严厉打击违建行为。剩余18条街路架空通讯管线全部改造落地。发挥数字地理空间框架项目功能，建设智慧城市。各县(市)也要加大城市建设和管理力度，为群众营造宜居生活空间。

(四)实施高纬寒地现代农业提升工程。发挥寒地生态资源优势，提高粮食产能和产品品质，构建优质安全农业体系。建基地。稳定优质大豆生产规模，扩大优质小麦种植面积，科学调控玉米、水稻等高产高效作物种植面积，绿色(有机)无公害农产品种植面积达到850万亩以上。新增北药种植面积15万亩、蓝靛果忍冬1万亩。建设非转基因大豆生产保护区和黑龙江流域高产试验区，现代农业示范园区达到50个，辐射面积300万亩。大力发展畜牧业，突出“两牛一羊一猪”，加快标准化、规模化养殖小区和牧场建设，推进嫩江万头奶牛现代示范牧场和百万头生猪产业化项目。积极发展水产业，力争省部级健康养殖示范场达到30处。育产业。依托地缘和资源优势，引进经营主体，做大做强农、畜产品深加工产业，扶持嫩江翔河油脂、鸿兴面粉等企业，重点抓好黑河中兴牧业、嫩江辰鹰乳业、孙吴爱俐华精制糖等项目建设，争取中兴牧业纳入全省现代奶牛示范场。大力发展现代林下经济，打造林菌、林果、林药、林业养殖、林业蔬菜、林业种植6个支柱产业。全市规模以上产业化龙头企业达到70家。完善利益联结机制，探索企业入股合作社、合作社自建企业、合作社与企业合作经营模式，落实订单面积720万亩。各级政府安排专项资金，解决农村合作组织资金周转问题。拓展局市共建领域，促进“三代”向全产业链、产加销全程合作延伸。打品牌。突出品牌整合，强化宣传推介，组织企业参加大型展会，扩大爱辉山珍、五大连池矿泉、大黑河岛冷水鱼、长乐山大果沙棘等特色产品知名度，叫响寒地绿色农业品牌。绿色(有机)认证产品达到70个。加强耕地保护和土壤改良工作。严格农产品质量安全监控，完善县级农产品检测站建设，加大农产品质量检测密度，建立生产记录、产地证明和产品准出追溯制度。

(五)实施对俄合作转型升级工程。发挥边境口岸优势，巩固对俄合作地位，形成全方位、宽领域、多层次立体交流格局，建设中俄友好合作示范区，打造中俄合作高地。提升进出口加工水平。坚持“出口抓加工、进口抓落地”，提高进出口商品落地加工率，实现过埠增值。扩大服装、果蔬、汽车、工程机械等大宗骨干商品出口，打造全省最大的对俄出口汽车机械制造销售平台，提高地工产品出口份额。增加电力、大豆、燃料油等资源能源类商品进口，拉动本港过货量。支持企业做大做强，跟踪服务重点外贸企业50家。深化对俄项目合作。实施“走出去”战略，支持企业参与俄资源能源开发，推进云津、首钢集团在俄开发大型铁矿煤矿，建设深加工基地。油品储运完成核准设计。阿州建筑装饰材料综合市场招商入驻。年产60万吨水泥粉磨站投产。促进境内外园区互动。加快中俄联合高新区建设，规划建设中俄国际科技产业城。加快北丰农业、和兴物流建材等境外园区建设，力争合作区阿州工业园区基础设施开工，构建内外互动、产业互补的跨境产业链。按照省探索建立对俄自由贸易区总体布局，积极做好争取工作。加强人文交流合作。完善与俄民间交往合作机制，筹建公共外交协会。巩固与阿州立法委员会、布市自治机构和企业家联谊会交流合作成果，深化与萨哈(雅库特)共和国合作。办好第五届中俄文化大集。与俄方联合举办大黑河岛国际经贸洽谈会和阿州展销会。做好“反法西斯战争”胜利七十周年纪念活动准备工作，筹建苏军援华抗日纪念馆。

(六)实施文化旅游精品工程。开展“旅游宣传促销年”活动，加强与媒体合作，扩大文化旅游影响力。加大对文化旅游产业扶持力度，设立旅游发展专项资金。旅游收入增长10%以上。培育精品文化。弘扬欧陆、民俗、红色、知青等地域文化，重点建设中俄民族风情园、北大荒知青产业园、黑河森林湿地博物馆、中国陆地边防馆、俄罗斯珠宝玉石交易城“两园两馆一城”，打造俄罗斯油画、冰雪画、中俄影视创作基地。加快北安新文化广场建设。各县(市、区)要立足特色，挖掘古驿站文化、火山文化、红玛瑙文化内涵，做大做强文化产业。打造精品线路。完善黑河—贝加尔湖生态之旅，开发黑河—布市乡村之旅和垂钓之旅，争取开通中俄跨境自驾游。探索俄籍中小客车进入互贸区。加快与珠三角、长三角及环渤海地区旅游线路对接。强化市域内旅游合作，实现资源共享、信息互送、市场互动，增加旅游收入。建设精品景区。加大五大连池风景区开发保护力度，做好世界地质公园和世界生物圈保护区评估工作，力争卧虎山景区和SPA城开工，完善新镇配套设施。加快五道豁洛旅游养生岛、新生鄂伦春族乡国际狩猎场、爱辉区和五大连池自驾车营地等项目建设，五大连池山口湖、孙吴胜山要塞等重点景区提档升级。加强旅游纪念品开发，延长旅游产业链。

(七)实施生态建设工程。坚持生态优先发展战略，认真实施大小兴安岭生态保护工程，完善资源有偿使用和生态补偿机制，保护蓝天碧水，建设美好家园。加强生态治理。加大水土流失治理力度，严格执行《水土保持法》，建立水土保持目标责任制，重点做好矿山、砂金过采区、江河源头流域的水土流失防控。抓住国家实施第二轮退耕还林政策契机，推进以“一退三还”为主要内容的退耕还林，实施“三北五期”工程。造林绿化36.6万亩，碳汇造林2500亩，抓好乡镇村屯、道路、工业园区、城市郊区造林绿化。实施森林改造培育工程，提高森林质量，中幼林抚育43.7万亩。全面禁止商业性采伐。积极申报国家木材战略储备基地。强化生态保护。实行最严格的生态保护制度，拒绝危害生态环境和有重大安全隐患的项目。高度重视森林防火工作，确保不发生重特大森林火灾。启动林地和森林、湿地、物种三条生态红线保护行动，严厉打击侵占林地、毁林毁草毁湿和破坏野生动物资源行为。林业有害生物无公害防治率达到81.5%以上。继续开展退耕还湿和补水保湿，重点保护一批典型湿地。争取公别拉河湿地自然保护区、中俄林业科技合作园区晋升国家级。办好第七届中俄林业生态建设学术论坛。抓好节能减排。提高清洁能源、可再生能源比重，严格控制高耗能、高污染项目准入，发展循环经济。全面推进工业企业、交通运输、公共机构、建筑等领域节能工作。提高污染防治水平，重点推进孙吴污水处理厂、嫩江和五大连池污水处理厂二期扩建等8个项目，逊克、五大连池供热企业脱硫项目。加强对重点行业和重点工程减排监管，将自动监控设施和中控系统建设运行纳入企业环保设施管理范畴。

(八)实施新型城镇化建设工程。坚持以人的城镇化为核心，以综合承载能力为支撑，探索符合黑河实际、具有地域特色的新型城镇化道路。推进产业强县。北安要发挥好我省正北部区域中心城市作用，发展壮大以商贸物流为主的现代服务业、绿色食品产业和新能源装备制造业，打造“老省会、新商都”。嫩江要大力培育矿产、食品工业等主导产业，争当县域经济排头兵。五大连池要加快培育矿泉产业和旅游经济。五大连池风景区要开发保护好世界级的旅游资源，打造“活火山观光旅游、冷矿泉康疗养生”胜地。爱辉区、逊克和孙吴要依托口岸优势，建设沿黑龙江开放带，发展对俄进出口加工、生态健康产业。打造特色城镇。做好新型城镇化规划布局，衔接好产业发展规划与小城镇建设规划，建设特色鲜明、环境整洁、生态环保新型城镇。培育优势产业，促进产城融合，打造罕达汽、多宝山、赵光等工业重镇，通北、龙镇等商贸大镇，新生、主星等旅游名镇，提高吸纳就业能力和辐射带动能力。推进15个新型城镇化试点乡(镇)建设，完善道路、供热、给排水等基础设施，增强教育、医疗、文化等公共服务功能。建设美丽乡村。遵循自然规律，充分挖掘历史价值、旅游价值和建筑价值，体现地域特色、民族特色和文化传承，实现布局与景观相协调，人与自然相和谐。集中打造黑龙江沿江带和嫩黑公路沿线新农村景观带，抓好30个重点村建设。推进基础建设和环境综合整治，抓好国省干道两侧乡村的道路建设、泥草房改造、房屋维修，努力建设生态宜居、聚商宜业、魅力宜游的生态人文美丽村庄。

四、扎实做好民生稳定工作

按照“守住底线、突出重点、完善制度、引导舆论”要求，做好民生工作，编织保障基本民生的安全网。突出抓好就业再就业。深入实施“双三零工程”，高校毕业生初次就业率达到80%。加强就业技能培训，就业培训1.35万人，创业培训2500人。城镇新增就业1.5万人，就业困难人员实现就业4000人，城镇登记失业率控制在4.6%以内。加快发展社会事业。办好人民满意教育，培养名优校长、名优教师，规范发展学前教育，均衡发展义务教育，突出抓好特殊教育，特色发展高中教育，保住优质学苗，资助贫困学生。规范办学行为，清理乱办班、乱补课。开展“提高医疗服务质量年”活动，狠抓医德医风建设。完善文化基础设施，文化馆(站)免费开放。深化“惠家工程”，做好人口计生政策调整准备工作。提升社会保障能力。不断扩大社会保险覆盖面，建立工伤保险费率浮动机制，继续提高社会保险待遇标准，市直城镇职工基本医疗保险住院医疗费最高支付限额由8万元提高到10万元，大病医疗补助由10万元提高到15万元。城市低保标准提高到每人每月376元，农村低保标准提高到每人每年2181元。新农合人均筹资标准提高到390元，参合率保持在99%以上。维护社会安全稳定。落实安全生产责任制，完善重大危险源安全监控系统，坚决遏制重特大事故发生。加强食品药品监管，建立全市统一的食品药品检验检测机构。完善行政复议案件受理审理机制，强化信访责任制，及时就地解决群众合理诉求。做好社区矫正工作。加强应急演练，提高应对突发事件、重特大自然灾害的处置能力。发挥边防委职能作用，建立联管联宣联评机制，加强国防教育，提高国防意识，确保边境安定。争创省级双拥模范城“四连冠”。

今年，市政府要集中办好“十六件民生实事”。一是改善住房条件，全市改造农村泥草(危)房6980户，恢复重建水灾损毁房屋355户。市区建设廉租住房150套。二是改善市民生活环境，市区改造旧住宅小区30个。改造城中村、烧火楼86户。改造老旧供热管网23公里，力争两年内将小区锅炉房全部拆除。三是方便市民出行，市区改造龙源路职教中心、龙滨路中龙市场附近等5处绿荫停车场，增加车位80个，逐步缓解市民停车难问题。安装公共自行车停放架60处。四是加大环卫投入，提高市区一线环卫工作人员工资水平和福利待遇。购置清冰雪专用设备，市区主要街路机械化清雪率达到100%。五是改善农村生活条件，全市新建农村饮水工程45处，解决2.2万人饮水不安全问题。六是加大教育投入，全市新建改扩建公办幼儿园9所，创建市级一类幼儿园6所、省级义务教育标准化学校8所、先进校3所。建设“安全和谐校园”，城内校园视频监控纳入公安机关监控系统。七是加快教育信息化建设，全市中小学宽带网络接入比例达到60%，30%班级实现“优质资源班班通”，15%师生实现“网络学习空间人人通”，市直及每个县(市、区)各建设1所信息化示范校。八是改善学校条件，全市城内学校和三分之二乡镇学校实现地面硬化，对现有29所农村学校“小火炉”进行改造。九是办好职业教育，加快推进黑河技师学院和省部共建国家现代农村职业教育改革试验区试点县建设。十是优化医疗资源布局，启动市第一人民医院易地新建工程。十一是改善医疗条件，安排财政资金，为市第一人民医院、市中医医院、市口腔医院购置先进医疗设备，建设远程会诊及远程医疗教育系统。医院视频监控纳入公安机关监控系统。十二是打造“动感城市”，开展群众性全民健身活动、青少年上冰雪活动。办好市第五届运动会。完成市体育场建设，力争体育馆主体工程完工。十三是优化口岸通关环境，旅检实行旅贸分开、客货分流，实行“无午休”、“无假日”工作制，开关时间与俄方同步。货检开通果蔬、鲜活产品“绿色通道”。十四是加大社区建设力度，全市城市社区达标率、农村社区启动率均达到80%以上，在社区内辟建冬季室内活动场所。城市社区和300平方米以上农村社区，全面推进居家养老服务。十五是推进市第二社会福利院扩建工程，打造异地养老服务示范基地。十六是推进市殡仪馆易地迁建、南山公墓扩建及公益性公墓征地工作。

五、全面深化重点领域改革

按照国家、省关于深化改革的总体部署，坚持先易后难、积极稳妥原则，把握节奏力度，对方向明确、具备条件的改革事项先行先试，统筹推进重点领域和关键环节改革。深化行政体制改革。研究制定政府“权力清单”和市场“负面清单”，厘清政府与市场、政府与社会边界。全面清理和减少审批事项，规范非税收缴，集中统一收费。推进市直“两集中、两到位”行政审批职能归并改革，部门审批事项整合集中后，进驻行政服务中心。推进新一轮政府职能转变和机构改革，加快事业单位分类改革。清理工商登记前置审批项目，放宽经营范围和场所限制。培育发展社会组织，改革登记管理制度，大力推进政社分开。深化预算改革，逐步推行部门预算公开。开展专项清查，盘活国有资产，推进公务用车和公务接待改革，降低行政成本。加快农业经营体系改革。改善乡村治理机制，增强农村发展活力。坚持家庭经营基础性地位，鼓励土地承包经营权向家庭农场、专业大户、农机专业合作社集中。尊重农民主体地位和愿望要求，发展农村合作经济组织，力争将符合标准的农机合作社，打造成省级规范社和示范社，建设管理规范、效益较高、带动力强的规模化合作社20个。创新新型城镇化发展机制。推进户籍制度改革，推行流动人口居住证制度，在投亲、投资、购房、人才引进等方面，最大限度放开户口准入。逐步建立城乡劳动者平等就业、同工同酬就业制度。完善城镇养老、医疗、失业保险等制度，推进城乡社保制度有效衔接，让农业转移人口真正融入城市。增强小城镇发展活力，对经济总量大、吸纳人口较多的城镇，适当下放与经济总量和人口规模相适应的经济社会管理权限。推进社会事业改革。创新人才引进机制，大力引进急需的矿产开发、城市规划和教育、卫生等领域高层次人才。突破校长、教师校际交流瓶颈，均衡配置教育资源。深化医药卫生体制改革，加快公立医院改革步伐，巩固完善基本药物制度和基层机构运行新机制。稳步推进国有文艺院团体制改革，引导和鼓励社会资本兴办文化企业。积极推进其他领域改革，充分释放发展活力。

各位代表，推进黑河发展，必须加强政府自身建设，努力建设为民、务实、清廉政府。要深入开展群众路线教育实践活动，对群众重大关切及时回应。多干打基础促发展的事，多干益长远利民生的事，多干增和谐保稳定的事。坚决贯彻落实市委决策部署，自觉接受人大法律和工作监督，接受政协民主监督，接受社会各界和人民群众的监督。坚持依法行政，推行政务公开，认真做好重大决策社会风险评估，提高科学决策、民主决策、依法决策水平。坚持带着问题下基层，带着感情见群众，听民意，解民忧。严格执行党政机关厉行节约反对浪费条例，认真贯彻国务院“约法三章”，不新建政府性楼堂馆所，不增加财政供养人员和“三公经费”。落实政府系统党风廉政建设责任制，防控廉政风险，强化对关键领域、重点项目、重大资金的行政监察和审计监督。

各位代表，繁荣的边疆、美丽的风光、漂亮的城市、幸福的人民，是全市上下的共同期待。我们要顺应全市人民对美好生活的向往，在省委、省政府和市委的坚强领导下，勇于担当重任，不负人民重托，为黑河的美好明天而努力奋斗！
